# Perceived Potential and Challenges of Supporting Coronary Artery Disease Treatment Decisions With AI: Qualitative Study

**DOI:** 10.2196/81303

**Published:** 2026-02-06

**Authors:** Khara Sauro, Bishnu Bajgain, Cody van Rassel, Bryan Har, Robert Welsh, Joon Lee

**Affiliations:** 1 Department of Surgery, Cumming School of Medicine, University of Calgary, Calgary, AB, Canada; 2 Department of Oncology, Cumming School of Medicine, University of Calgary, Calgary, AB, Canada; 3 Department of Community Health Sciences, Cumming School of Medicine, University of Calgary, Calgary, AB, Canada; 4 Data Intelligence for Health Lab, Cumming School of Medicine, University of Calgary, 3280 Hospital Dr NW, Calgary, AB, T2N 4Z6, Canada, 1 403-220-2968; 5 Department of Cardiac Sciences, Cumming School of Medicine, University of Calgary, Calgary, AB, Canada; 6 Faculty of Medicine and Dentistry, University of Alberta, Edmonton, AB, Canada

**Keywords:** coronary artery disease, clinical decision support, artificial intelligence, technology adoption, implementation science, stakeholder engagement

## Abstract

**Background:**

Coronary revascularization decision-making for patients with coronary artery disease (CAD) can be complex and challenging. Artificial intelligence (AI) has the potential to improve this decision-making by bringing data-driven insights to the point of care.

**Objective:**

We aimed to elicit, collect, and analyze various stakeholders’ perceived potential and challenges related to developing, implementing, and adopting AI-based CAD treatment decision support systems.

**Methods:**

A facilitated small-group discussion method, known as a World Café, was conducted with general cardiologists, interventional cardiologists, cardiac surgeons, patients, caregivers, health system administrators, and industry representatives. One-on-one interviews were conducted for participants who could not attend the World Café. Perceived potential and challenges of AI-based CAD treatment decision support systems were solicited by asking participants three broad questions: (1) What is most challenging about revascularization decision-making? (2) How could an AI tool be integrated into the existing clinical workflow? (3) What are the critical components that need to be considered when developing the AI tool? Thematic analysis was performed to identify themes from the data.

**Results:**

Nine participants completed the World Café, and 3 participants completed the one-on-one interviews. Five main themes emerged: (1) evidence-based care, (2) workload and resources, (3) data requirements (subthemes: patient-centered approach, evidence-based AI, and data integration), (4) tool characteristics (subthemes: end user built; generation and presentation of decision support information; user-friendliness and accessibility; and system logic, reasoning, and data privacy), and (5) incorporation into clinical workflow (subthemes: AI as an opportunity to improve care and knowledge translation).

**Conclusions:**

While health care providers aim to provide evidence-based care, CAD treatment decision-making can often be subjective due to the limited applicability of clinical practice guidelines and randomized controlled trial evidence to individual patients. AI-based clinical decision support systems may be an effective solution if the development and implementation focus on the issues identified by end users in this study (patient preference, data privacy, integration with clinical information systems, transparency, and usability).

## Introduction

Coronary artery disease (CAD) is characterized by reduced blood flow to the heart muscles caused by plaques in the coronary arteries. The gold standard diagnostic procedure for CAD is coronary angiography performed in cardiac catheterization laboratories, using radiocontrast agents and x-rays to diagnose the disease. Typically, the treatment decision involves determining whether the problematic coronary arteries need to be revascularized via either percutaneous coronary intervention or coronary artery bypass graft (CABG) surgery, or whether the most appropriate treatment is medical therapy only.

While clinical practice guidelines based on randomized controlled trials exist [1], coronary revascularization decision-making can be complicated by complex CAD (eg, multivessel disease), challenging coronary anatomies, comorbidities, unique patient characteristics, and patient preferences. Although multidisciplinary Heart Team approaches, where diverse specialists, including general cardiologists, interventional cardiologists, and cardiac surgeons, discuss the patient case and formulate the best treatment as a group, are recommended for revascularization decision-making for complex CAD [2,3], they are neither standardized nor evidence-based, making it difficult to operationalize complex treatment decision-making systematically.

Artificial intelligence (AI) has the potential to support coronary revascularization decision-making via data-driven insights. By leveraging patterns and relationships learned from a large amount of patient data, AI models can generate and present personalized decision support insights at the point of care. However, even if AI models with good performance are available for deployment, their technical and operational implementation in real-world clinical environments remains challenging and requires adoption from a variety of stakeholders, including patients, clinicians, health system administrators, health care payors, researchers, and developers [4]. Understanding the barriers and enablers to adopting AI-based clinical decision support systems (CDSS) is critical to developing and implementing such systems in clinical practice.

This study aimed to understand the perceptions of how an AI-based CDSS can facilitate CAD treatment decision-making.

## Methods

### Study Design and Setting

A World Café [5] was used to elicit and collect stakeholder perceptions about the use of an AI-based CDSS for CAD treatment decision-making. A World Café is a formal, semistructured method that engages diverse stakeholders through multiple rounds of small group discussions, each guided by a targeted question. It is designed to create an open, café-style atmosphere that encourages equitable participation and the flow of ideas across groups. This method enables intimate discussions among participants with varied perspectives, supporting the identification of themes relevant to a topic.

This study intended to complete a single World Café; however, some of our key end users (clinician participants) were unable to attend the World Café due to unexpected urgent patient cases. To ensure that these participant perspectives were captured in the dataset, 3 additional one-on-one interviews were conducted at a later date using the same semistructured protocol and targeted questions used in the World Café.

The World Café and interviews took place in Alberta, Canada, from May 2, 2022, to July 18, 2022, using the videoconferencing and online meeting platform, Zoom (Zoom Communications). Only participants and researchers were present during the meetings and interviews.

### Participants and Recruitment

Study participants were recruited in Alberta and included: (1) clinicians involved in the care of patients with CAD including general cardiologists, interventional cardiologists, and cardiac surgeons; (2) health system administrators; (3) private-sector representatives from the cardiovascular information system industry; and (4) patients and caregivers (individuals aged 18 y and older with CAD, or caregivers supporting individuals with CAD).

All clinician participants were practicing physicians within Alberta Health Services (AHS), which is one of the largest fully integrated provincial health systems in North America. AHS oversees centralized delivery of acute care, emergency medical services, diagnostics, and many community-based programs for over 4.4 million Albertans. Although Alberta operates within Canada’s universal, publicly funded health care system, its structure differs from many jurisdictions in Canada and abroad by unifying services under a single provincial authority rather than regionally or privately administered systems [6].

Participants were identified using purposive sampling to maximize variation in backgrounds and sex differences. Potential participants (World Café participants [WCPs] and one-on-one interview participants [OIPs]) were recruited using our research network and invited to participate via email. Once potential participants indicated an interest, the consent script was sent to them via email. They were provided with multiple opportunities to ask questions before completing the oral consent process. The consenting process occurred before the World Café and one-on-one interviews.

### Data Collection

Consistent with the World Café methodology [7], data were collected through facilitated discussion on broad questions. Three questions were posed to both the WCPs and OIPs sequentially: (1) What is most challenging about revascularization decision-making? (2) How could an AI tool be integrated into the existing clinical workflow? (3) What are the critical components of the AI tool that need to be considered when developing the tool?

A facilitator guided the discussion and used prompts to generate discussion for each question. A note-taker collected field notes to document the context of the discussion (eg, the physical environment and individuals’ nonverbal communication) and captured a summary of the discussion, which was shared with the participants at the end of the session (member checking). The World Café session and interviews were digitally recorded and transcribed verbatim, and the field notes were incorporated into the transcripts for analysis.

### Reflexivity

The World Café and interviews were conducted by trained, experienced male and female facilitators who had formal graduate-level and experiential training in qualitative methodology and interview facilitation. Several members of the research team, including the facilitators and principal investigators, had pre-existing professional and nonprofessional relationships with some participants, which may have influenced rapport and data interpretation. One principal investigator was a family member of a patient with CAD who had recently been diagnosed and had an urgent CABG. During the analysis phase, these pre-existing relationships and researchers’ personal backgrounds were explicitly discussed during team meetings to reflect on how personal experiences, disciplinary backgrounds, and expectations may shape data interpretation and the construction of final themes.

### Data Analysis

Transcripts from both the World Café and one-on-one interviews were uploaded, managed, and analyzed using NVivo (version 12.0, Lumivero). The Clarke and Braun [8] approach to thematic analysis was used to analyze the data. An inductive approach was used to identify codes and themes from the data. A first analyst familiarized themselves with the data and identified and established codes in a coding book as a reference. A second independent analyst then familiarized themselves with the data and reviewed the preliminary codes identified by the first analyst, revising and adding new codes while interpreting the data as a circular process that moved back and forth between smaller parts of the transcript and the whole text. This iterative coding process was applied as new themes emerged, and the transcripts were reread to verify that the codes and themes were not missed. These coding “nodes” were discussed among the research team and then consolidated into themes [8]. Coding discrepancies between analysts were addressed through discussion and joint review of the relevant transcript segments. Consensus was achieved through iterative comparison of interpretations, and any disagreements were resolved collaboratively to ensure consistent application of codes across the dataset.

In contrast to the group dynamic characteristic of the World Café, individual interviews may elicit more detailed and individualized reflections. To reconcile these methodological differences and enable direct comparison between formats, all transcripts were coded using the same unified coding framework. Codes and themes were examined for convergence and divergence, and only themes supported by patterns across both data sources were used in the final analysis. Although the one-on-one interviews were conducted following the World Café, the interview guide was not refined or modified based on World Café findings, and the same semistructured protocol was used across formats.

### Trustworthiness

Various strategies were used to ensure the trustworthiness of the findings [9]. The transcripts were reviewed by the World Café and interview facilitators for accuracy before analysis. We used member checking at the end of the World Café and each interview by summarizing the discussion and asking participants if we accurately captured the discussion. Regular peer debriefing and discussion took place between members of the research team about the representation of this study’s population, recruitment, data collection strategies, and data analysis, from the data coding process to the emerging themes, to enhance the accuracy of the results. The results were reviewed and refined by all authors, some of whom were participants.

### Ethical Considerations

The University of Calgary Conjoint Health Research Ethics Board approved this study (REB20-1879). Before participation, explicit oral informed consent was sought and obtained from all study participants. The privacy and confidentiality of participants’ data and identity were maintained by following the approved research data security and privacy protocol. Participants were not compensated. Additionally, this paper follows the COREQ (Consolidated Criteria for Reporting Qualitative Research) guideline (Checklist 1) [10].

## Results

### Overview

The World Café was conducted on May 2, 2022, and the interviews were conducted between June 15 and July 18, 2022. The World Café lasted about 120 minutes, and each interview ranged from 45 to 60 minutes. The participants (9 male; 3 female) were cardiologists (n=4), interventional cardiologists (n=2), cardiac surgeons (n=2), health system administrators (n=1), patients and caregivers (n=2), and an industry representative (n=1). Clinician participants (n=8) included early-career (n=3), middle-career (n=4), and senior professionals (n=1).

Five overarching themes emerged from the data: (1) evidence-based care, (2) workload and resources, (3) data requirements (subthemes: patient-centered approach, evidence-based AI, and data integration), (4) tool characteristics (subthemes: end user built; generation and presentation of decision support information; user-friendliness and accessibility; and system logic, reasoning, and data privacy), and (5) AI incorporation into clinical workflow (subthemes: AI as an opportunity to improve care and knowledge translation). Each theme is described in detail in the ensuing sections, with example quotes tabulated in Tables 1-5 for each theme. Quotations are identified by stakeholder group and by data collection source (ie, WCPs or OIPs).

**Table 1.** Example quotes related to evidence-based care (theme 1).

Description	Example quotes
The importance of evidence-based clinical practice.	“We need more evidence when the areas we do have evidence still aren't standardized… Could more evidence help standardize things? And I do want to say that there are areas, certainly, that we need more evidence, but just like the counterbalance, there is, there are areas where we have evidence, and it still hasn't standardized practice.” [OIP #1, surgeon] “It certainly does. And I think it would for everybody. Everybody should be thinking of the guidelines, but it's kind of a starting point because it's often the nuances, or there's the other clinical variables that aren't in the classic guidelines that are important considerations.” [OIP #3, interventionalist] “It can be challenging identifying which patients you're going to want to proceed with revascularization versus proceeding with medical therapy… it does become somewhat knee-jerk that a person has in anginal symptoms or they have a non-invasive test that's suggestive of ischemia, and automatically they get sent to the cath lab with the thought that they're going to be revascularized. Now, the existing literature and the existing guidelines don't actually support that… it can be challenging…” [OIP #2, interventionalist]
Uniform and standardized decision-making between clinicians: different priorities based on their values & success rates (PCI^a^ vs CABG^b^).	“I think most decisions for revascularization are made ad hoc, on the spot, and I think that's reasonable for most of the time, but it really is the setup for a practitioner dependent practice… I think all of us see variation in practice, and I think all of us see that there's areas that aren't standardized… It would be nice if things were standardized, it simplifies things and allows everyone to ensure that we're aligned or at least the expectations are clear.” [OIP #1, surgeon] “I think there is variation in practice. But, say, between different surgeons, there's definitely variability. But, ultimately, say, if it's in the middle of the night, then it's basically what my preference or opinion is, I guess, at that point. What my colleagues might do might be different, but it doesn't really affect my decision process at that point.” [OIP #3, interventionalist]

a PCI: percutaneous coronary intervention.

b CABG: coronary artery bypass grafting.

**Table 2.** Example quotes related to workload and resources (theme 2).

Description	Example quotes
The impact of decision-making process due to workload and physician burnout. Lack of time to explore and discuss patient information due to high volume of work.	“When I have been burnt out, of course that impacts my practice, of course that impacts my decision making. I imagine that each of these... I think there's a very high resiliency rate within each of these groups, and I think, a lot of self-awareness to monitor burnout. It's impossible for me to quantify the impact, I'd just be speculative, but I think that all of us have to be mindful of that impact in decision making.” [OIP #1, surgeon] “When we're burnt out and we're overloaded sometimes, there's a tendency towards the path that's going to give us a more definitive answer more quickly.” [OIP #2, interventionalist] “The other considerations are the timing of revascularization. Is there active ischemia at the time? Is it an emergency that needs to be done right away? Or is it something that can wait until something else is optimized, either antiplatelet strategies or anticoagulation or other patient variables? Clinical status? Timing on like ... Is it emergency or not? Or is it urgent? Or is it elective?” [OIP #3, interventionalist] “I was thinking about time as being one of the biggest issues for me and also acuity. So, I find that you have more time to weigh those risks and benefits and do your own cost-benefit analysis in the more stable scenario. But, in the acute phase, that time to do that goes at the side of trying to get the intervention going and the case started… It should be standardized for all patients, regardless of the presentation. But in my own individual experience, I find that time is really an issue, and it depends on the urgency of the scenario.” [WCP #6, cardiologist] “One is synthesizing all of the data that comes in. So, whether it's the anatomy, the patient comorbidities presentation, and what everyone's perspective on feasibility of getting a good result, whether it's by angioplasty or bypass surgery. So, I think it's putting everything together… you're trying to get through all of these patients, being comfortable that you've gathered all the relevant data to make the right decision is quite difficult…” [WCP #5, interventionalist] “We might get a mailing list the day before…, you might spend a little bit of time in the evening reviewing the angiogram films. And the next day they might only spend five minutes on a patient… So, you're trying to get through all of these patients, being comfortable that you've gathered all the relevant data to make the right decision is quite difficult, given the fact that a lot of times you have never met the patient… So, certainly that you can get time pressure and not discuss patients thoroughly enough.” [WCP #5, interventionalist]
Considering cost of care (PCI^a^ vs CABG^b^) vs value of care.	“Back to our patient's perspective, I think we do look at risks of the non-discussed pieces of it when we make our decisions with patients. So, obviously as clinicians, we talk about death or MI stroke, but there are a lot of other things that go into the decision-making like time in hospital, recovery status… we would want to discuss with the patient to make sure they understand. So, at 10 years you may have a slightly better risk of death, but in the meantime, you've got a recovery period that would be hard to manage. So, cost may be less so than the patient discussions, but as the healthcare system tightens, cost will become more and more important in the future.” [WCP #4, interventionalist] “We do talk about value, defining value as outcome over cost. So, it's not that the cost doesn't matter. And I think the system is willing to pay the cost as long as the outcome achieved from that cost makes sense. So, I think in our system, we would talk more about value” [WCP #3, administrator] “When you're pressed for beds if the results are close enough between bypass and PCI, does your resource limitation push you more towards one or the other?... And so, certainly we did have those discussion... you just deal with one or the other either with surgery or angioplasty, then the fact that five years down the road they might be back for a second procedure. But you just try to keep the resources freed up in the system, whether it's time or money or beds.” [WCP #5, interventionalist] “Everything that we're talking about is incredibly expensive… I would think, in the scheme of budget for this, this would be relatively small, and anything that makes things more efficient probably will save money more than whatever it costs.” [OIP #3, interventionalist]
Considering resources (e.g., cost, investment), and willingness to invest and implement the AI^c^ tool.	“If we want these tools to be adopted, they have to be purchased… a lot of the times we are making those arguments as a return on investment, essentially saying that if they pay this much for this software, it will save them time, it will lead to improvements in quality for value-based reimbursement or these sorts of things… So, I do think there's the clinical perspective in terms of, directly with the patient and beds and everything. And then there's also the administrative perspective around time and resources and whether they are willing to put in the effort to implement this in practice in order to actually see the results. I think it's an important consideration, even this early on in the process.” [WCP #9, industry partner]

a PCI: percutaneous coronary intervention.

b CABG: coronary artery bypass grafting.

c AI: artificial intelligence.

**Table 3.** Example quotes related to data requirements (theme 3).

Subtheme and description	Example quotes
Patient-centered approach	
Complex patients with multimorbidity	“I think one of the bigger challenges that's happening more and more now is our patients are older. They're more complex. They have more comorbidities. The risks of everything are higher. Their disease is getting more complex. There are sometimes not reasonable PCI options… And so, you are not infrequently trying to treat a patient who has complex multi-vessel disease, who is a poor candidate for surgical revascularization, or a very high-risk candidate for surgical revascularization, who is equally a high-risk candidate for percutaneous revascularization.” [OIP #2, interventionalist] “There are the anatomic things like the coronary anatomy. There are the other comorbidities are a big role... I guess the factors that relate to their potential benefits. Are they potentially receiving a symptom or a survival benefit? And then what are their risk factors?... Because it's always a balance of benefit versus risk.” [OIP #3, interventionalist]
Understanding patient expectations and preferences	“If you give the patients a choice, they'll all pick PCI. And PCI is great for a lot of them, but it's not right for everybody. And that's where that education has to come in, and then they can make their decision.” [OIP #3, interventionalist] “I chose a course of treatment that had the lowest chance of incontinence over the others. So, it's weird, I'm not sure patients really think exactly like clinicians in this situation. I was opting for quality of life over other factors, and it served me well… But those would be important to me.” [WCP #2, patient] “Nobody wants to have surgery, but people are interested in their long-term outcomes. They don't want repeated heart attacks… the second one is also understanding what the patient's preference is and how much of that weighs on the decision. So, if it's 60% in favor of bypass and 40% in favor of angioplasty from a clinician standpoint, but what if the patient feels very strongly that they want to have angioplasty and you would need to tell them various substantial risk… or substantial benefit of bypass surgery. So, understanding what the patient preference is based upon what would happen if they were presented with the data.” [OIP #3, interventionalist]
Evidence-based AI^a^	
Representation of diverse patients in the data used to develop the model	“I think that making sure that you want the largest pool of data possible, but making sure that data represents a wide cross-section of demographics, and that you're not trying to ultimately end up generalizing it to populations that haven't been involved in generating the model. That's really important.” [OIP #2, interventionalist]
Complementing and improving upon the existing clinical practice and evidence	“I would love to see both strengths of AI put to work… there are a lot of things we know, and honestly, I think we're maybe not that bad at this, but it would be great to see if AI comes up with information or predictors that we didn't know, which would be the real beauty of this model.” [WCP #4, interventionalist] “You're going to do the standard stuff that we all think about, age, diabetes, hypertension, dyslipidemia, frailty, down the list, but the black box approach where you basically ask the computer to tell us what's in the model, I think would be very interesting and potentially the validation moving forward would be very exciting.” [WCP #4, interventionalist] “AI-based technologies is to be able to give us information that some standard studies don't give us… It may be possible that by applying more complex machine learning, you might actually be able to find an answer that we haven't been able to find so far in our existing evidence. So, I think that you have to be aware of what the existing evidence is, but if you find a different answer using new technology, well, I don't think you adopt it wholeheartedly and ignore what we have figured out already, but I think you need to integrate that in.” [IP #2, interventionalist] “The evidence is coming typically from trials that have a lot of nuances to them. Applying guidelines or a specific trial to a specific patient can be real challenge because they don't always directly apply. That's where the clinical judgment and oversight and gestalt, I think, play a role and is trying to say, well, the evidence that we have ... How well does it apply to this patient? And that's where they don't always directly apply.” [IP #3, interventionalist]
Data integration	
Ethics, privacy, and confidentiality	“I think that from a patient privacy perspective, that's going to be a really important thing to have a really good grasp on the ethics before that happens, because machine learning can be used to generate all sorts of models for all sorts of risks. And whether those things in the future are going to... Say, my EMR is plugged into machine learning, and it's able to automatically generate a detailed risk for, for cardiac death over the next 10 years, is that going to affect my patient's ability to get insurance? So, there needs to be a consent aspect in there for sure.” [OIP #2, interventionalist] “Obviously, confidentiality and privacy are massive when it comes to medical information. It certainly would have to be secure.” [OIP #3, interventionalist]
Integration with the EMR^b^.	“Something that can be incorporated directly into the EMR, so you can just say, ‘Oh, well, I want to calculate the whatever score for this patient,‘ so you can click on it and it can pull in whatever data that it needs. I think that the simpler you make it, the more likely you're going to see uptake.” [OIP #2, interventionalist] “When we talk about how this could be integrated in the existing workflow, I think we have to consider Connect Care as part of this…, Healthcare institutions have put significant investment into the implementation of electronic medical record systems… which opens up an opportunity for us, if we can integrate with those systems and make it fairly seamless experience.” [WCP #9, industry partner]
Integration of comprehensive data elements	“The critical components one is, as everyone's mentioned, is the APPROACH CARAT diagram and all the different components of it, whether it's the anatomy or the jeopardy score, lesion characteristics, previous stents, the comorbidities that are the classic comorbidities, whether it's renal failure or diabetes, left ventricular dysfunction, patient age, BMI, whether it's super high or super low. The ones that are harder to capture, I think, beforehand… other one is frailty… it's something that's really hard to capture, but I think the frailty piece is really important.” [WCP #5, interventionalist]

a AI: artificial intelligence.

b EMR: electronic medical record.

**Table 4.** Example quotes related to tool characteristics (theme 4).

Subtheme and description	Example quotes
End user built	
Engaging end users throughout the development and implementation processes	“When developing something that is intended to be used as a tool for someone, that person is the stakeholder and they should be engaged and heavily involved in all aspects of the development, training, implementation, and subsequent follow up and iterations. It's hard for me to imagine a step a clinician shouldn't be involved…” [OIP #1, surgeon] “Physicians and patients would be the end-users for those models that are generated. So, matter of asking what more information the end-users actually need.” [OIP #2, interventionalist] “I think something like this could be complicated enough that, without a physician or surgeon or an interventionist… clinical feedback would be important in order to tailor it to what the clinicians need to see.” [OIP #3, interventionalist] “What data are available, what data are easy to get, which ones would require the physician to do extra steps? And maybe prioritizing ones that are accessible or prevalent versus ones that might require extra work and therefore prevent adoption. So, I think that's, figuring out what we can do with Connect Care or very similar systems is important here.” [WCP #9, industry partner]
Engaging patients throughout and capturing patients’ voices	“If the AI tool was developed with the patient-centered focus with patient researchers actually involved in developing the tool. And it might provide a communication roadmap for some of your colleagues… and coach them in both the language and what the patient is actually looking from their clinician to provide in the way of information, so that the discussion is actually much more robust.” [WCP #4, interventionalist] “Asking the patients how they're doing, their mobility, quality of life, frailty, we know from other research that's been done that PROMs are quite predictive of outcomes, and they come out, when you run the AI with all the different features, PROMs often come out as contributing to that final prediction.” [WCP #9, industry partner]
Family physicians as potential end users	“Is that possible that it could be starting with a family physician instead of specialist cardiologist? I don't know where else it potentially could start. That way, it's an easier conversation for the family physician to have with the patient.” [WCP #1, patient] “I would fully agree with that (integrating AI in PHC)… The only thing is that some family physicians are overwhelmed by the breadth of knowledge they need to know about your diabetes, your heart's arteries, your medical therapy, and they really look to the cardiologists and the surgeons, where appropriate… the intricacies of decision-making.” [WCP #4, interventionalist]
Generation and presentation of decision support information	
Need for scores as a way to summarize granular information for easy interpretability and communication	“Making decisions about revascularization would be the development of more complex risk-prediction models… through machine learning, there would be the ability to generate a more sophisticated model for helping to estimate risk. And again, you're not going to ultimately have an AI making the decision about whether or not you're going to proceed with revascularization, but to be able to go into an interaction with a patient and be able to give them more granular information about what their risks are, that could be helpful.” [OIP #2, interventionalist] “I think it'll be determining what all risk factors need to go into this tool (AI) and what weight are you going to give to each risk factor and does presentation…, How do you give mobility a number, and comorbidities… but some of these risk factors are going to be a little bit difficult to try to quantify.” [WCP #7, cardiologist] “My first thought was on the APPROACH diagram, there's a jeopardy score and the jeopardy score is used, to a degree, to guide our revascularization decisions. It would almost make sense that this prediction tool puts some sort of either score or recommendation was my first thought.” [WCP #4, interventionalist] “Overlay how AI has actually helped populate the data in the various columns of chart data. That, to me, I think would be perhaps a good marriage of data… on the various success factors that you're striving towards by each treatment option. And then that would provide a good discussion basis for the physician and the patient and their family… if you stack them all together, you would have what would logically be the optimal treatment plan, just because the higher score and whatever it would add up, it would be ably demonstrated… it's easy to explain at the bedside, I think would be a big benefit rather than talking in clinical jargon.” [WCP #2, patient]
Importance of data quantity and quality	“I think that's how you would harness the true power of AI and machine learning. As much data as you could get in, I think that's how you could really…harness the power of this method.” [OIP #1, surgeon] “In order to get high quality information out of any machine learning system, it's entirely based on the volume of data you're able to provide it… I think the broader the data you're able to plug into a machine learning system, the better, because one of the big issues that I'm aware of with machine learning is just the impact of the bias of the information that you put in.” [OIP #2, interventionalist] “As a surgical resident who's doing a lot of research with clinical data, being able to extract that data efficiently would be key... I think we've struggled a little bit with the comprehensiveness of data and the efficiency of it.” [WCP #8, surgeon]
User-friendliness and accessibility	
Importance of an easy-to-use, intuitive user interface and automation.	“User interface are really important… I'm not still clear how to evaluate an algorithm… I don't have a good sense as to how to actually perform due diligence on a product or algorithm. And in the absence of the ability to do that, it's very difficult to know how much weight you would put on the response from an algorithm. So, I think that a bit of knowledge translation, or trying to validate an AI model in a manner that's understandable to clinicians is critical.” [OIP #1, surgeon] “I think a lot of what it comes down to is really just convenience, and speed, and ease of use… certainly, automated measurements, automated tracing of the left ventricle and calculation of, for example, a 3D left ventricular ejection fraction. These are areas that accuracy is improved… so I think usability becomes really, really important, particularly at a time when technology has in certain areas of medicine, led to a lot of added complexity with an unclear value proposition. And so I think that really providing an easy to use interface and easily digestible material is our answers, is really helpful.” [OIP #1, surgeon] “When I think about kind of AI tools, they're going to be things that physicians reach for when they want them… you're going to be reaching for every patient… for something as simple as like MDCalc to put in someone's Framingham risk score. So, you would want something that is easily accessible, something that is smartphone-based, or even something that can be incorporated directly into the EMR, so you can just say, ‘Oh, well, I want to calculate the whatever score for this patient,‘ so you can click on it and it can pull in whatever data that it needs. I think that the simpler you make it, the more likely you're going to see uptake” [OIP #2, interventionalist] “In general, surgeons aren't very technically or at least computer savvy. The simpler, the better, for sure. Because not too many surgeons will commit to a lot of learning for something like that, computer work, or have a lot of time to commit to it. Actually, it is quite important that it's something that's intuitive or easy to work with. Being user friendly, for sure, is important. Being accessible. Something that we can use remotely is important because that's when, I think, it'd be pretty useful. And then just current, I guess. Data or information that's real time.” [OIP #3, interventionalist]
System logic, reasoning, and data privacy	
Transparency and accountability	“I think that there does need to be some sort of transparency in how the tool is working… For decisions, an overall gestalt is used a lot... is this person a candidate for this? Is this person ... Would it be ... Would they do well with this? And so, the gestalt is an important part. With artificial intelligence, I don't know if gestalt is a part of it. That would be a challenge, I would think. Having some transparency, though, in how the algorithms or whatever it is that are making recommendations or decisions or things like that ... A transparency so that people can see how this tool comes to these conclusions.” [OIP #3, interventionalist] “I think the only thing that we have to be fully aware of and cognizant of… from a medical legal point of view, having a statement that X is the desired outcome without all of the qualifying pieces, if the Y procedure is done and the patient has a bad outcome, which may or may not be predictable, usually not predictable, it may lead to a medical legal nightmare… So, we just have to be cognizant of that and decide where this decision tool lands in terms of its availability and to who.” [WCP #4, interventionalist]
Living documents and real-time feedback	“From a workflow perspective, AI really needs to be almost synchronous with the test itself and providing real time feedback or near time feedback to be really clinically useful.” [OIP #1, surgeon] “I think it needs to be part of the living document and available at the time we do the angiogram to be most valuable. I would say that 90% of the time the revascularized option, which is anatomically best for the patient and comorbidly best for the patient, taking their patient profile is obvious 90% of the time. It's either a straightforward stent, we deal with it, move on. It's either ongoing medical therapy, we optimize that and move on, or it's a clear-cut patient that should be moved forward for bypass...” [WCP #4, interventionalist] “Being user friendly, for sure, is important. Being accessible. Something that we can use remotely is important because that's when, I think, it'd be pretty useful. And then just current, I guess. Data or information that's real time.” [OIP #3, interventionalist]
Data privacy and confidentiality	“I think that from a patient privacy perspective, that's going to be a really important thing to have a really good grasp on the ethics before that happens, because machine learning can be used to generate all sorts of models for all sorts of risks. And whether those things in the future are going to... Say, my EMR is plugged into machine learning, and it's able to automatically generate a detailed risk for, for cardiac death over the next 10 years, is that going to affect my patient's ability to get insurance? So, there needs to be a consent aspect in there for sure.” [OIP #2, interventionalist] “Obviously, confidentiality and privacy are massive when it comes to medical information. It certainly would have to be secure.” [OIP #3, interventionalist]

**Table 5.** Example quotes related to AI^a^ incorporation into clinical workflow (theme 5).

Subtheme and description	Example quotes
AI as an opportunity to improve care	
Considering AI as an opportunity in the existing clinical workflow.	“For sure, there's many opportunities… I think risk stratifying the lesion or identifying high risk lesion characteristics… identifying a culprit lesion, the predicted success of revascularization or subsequent stent complications. In another way of saying that would be determining what method of revascularization. I think those are all-important real-time feedback that an operator could receive… It would be really nice to pair that with non-invasive cardiac diagnostics, which would include echo, MRIs… Those are all areas that I think will be very, very interesting.” [OIP #1, surgeon] “I think that having some artificial intelligence to make suggestions is helpful. And like I said, to provide supporting information like risk profiles and stuff like that, but I think that ultimately, the conversation between the physician and the patient is always going to be a pretty big driving force for what path we go down.” [OIP #2, interventionalist]
Knowledge translation	
The importance of knowledge translation among end users.	“One of the challenges for us in knowledge translation here, trying to translate something that's extremely technical. And in fact, we're front-runners in the industry into something that's useful for both patients and caregivers. So, I don't think it's a game stopper, it's just something we have to be aware of and be knowledgeable about.” [WCP #2, patient] “You guys do such important work, really the patient should be knowing this stuff, like what's going on with their body at a doctor's visit, at a family physician. So, I think if this information's being shared.” [WCP #1, patient] “I don't have a good sense as to how to actually perform due diligence on a product or algorithm. And in the absence of the ability to do that, it's very difficult to know how much weight you would put on the response from an algorithm. So, I think that a bit of knowledge translation, or trying to validate an AI model in a manner that's understandable to clinicians is critical.” [OIP #1, surgeon]

a AI: artificial intelligence.

### Theme 1: Evidence-Based Care

#### Overview

All participants emphasized the importance of evidence-based guidelines to minimize variation in care and unify the Heart Team that makes treatment decisions for patients with CAD. The clinician participants explained that their clinical practice was based on their clinical knowledge and experiences, as well as existing medical evidence (including clinical practice guidelines). They stated that although there are some clinical guidelines, many are of low quality and endorsed the need for better guidelines. They also noted that while evidence and experience are foundational, CAD treatment decisions tend to be biased by the opinion of a single clinician and that each clinician has different priorities and experiences.

The clinician and health system administrator participants noted that, in addition to the evidence informing treatment decisions, factors such as the urgency of the case, provider workload, patient preferences, and time of day also influence treatment decisions. This led to comments related to timely and complete patient data (medical history and comorbidities), which is discussed in greater detail under theme 3 (data requirements).

#### Contrasting Perspectives

While all participants endorsed the importance of evidence-based care, clinicians primarily framed the issue as a challenge of guideline clarity and individual bias. In contrast, it was suggested that administrators may instead focus on the broader system-level influences that contribute to variability in revascularization decisions (explored in further detail in theme 2).

### Theme 2: Workload and Resources

#### Overview

Most clinician participants stressed that time is a significant issue for making treatment decisions around revascularization. They stated that due to the high volume of cases, there is minimal time available to comprehensively review patient information and discuss the patient. This challenge, combined with the urgency required to revascularize patients with CAD, makes it difficult to fully assess all the risks and benefits of each treatment approach and consider patient preferences. All participants endorsed the challenges related to the current strain on the health care system and clinicians.

There was also discussion about physician burnout due to persistently high workloads. Clinician participants expressed that when they are overloaded and burnt out, there is a chance they will make a treatment decision more quickly, without fully considering all factors.

Participants suggested that having access to all relevant information for making treatment decisions in one, easily accessible spot would facilitate decision-making, which might help to improve patient outcomes and resource use. This is further discussed in theme 5 (AI incorporation into clinical workflow). Further noted by the patient participants was the importance of also considering patient preferences, which is discussed under themes 3‐5 below.

Furthermore, there was some discussion about the cost of care vs the value of care (outcomes). Most of the clinician participants emphasized that they valued results over cost when making decisions about revascularization. However, they did suggest that long-term resource consumption should be considered before making treatment decisions, provided the evidence for effectiveness is comparable (eg, CABG vs percutaneous coronary intervention). The health care system administrator also discussed the issue of the value of care, especially in the current context of a resource-strained health care system.

Considerations of the cost and resources of implementing a new AI system into clinical workflow were discussed. It was noted that adopting AI in the clinical workflow is expensive and needs investment (time and money). However, participants suggested that the investment in AI may be relatively small if it eventually saves time and improves the quality of care. The industry participant expressed the importance of engaging and understanding health system administrators’ willingness to invest (time and resources) and implement the AI technology into practice from the early stages of the development process.

#### Contrasting Perspectives

Although all participants acknowledged the strain created by limited time and resources, clinicians emphasized how high workload, case urgency, and burnout directly affect their ability to thoroughly review patient information and weigh treatment options. Patients highlighted the importance of ensuring that their preferences are considered despite time constraints, whereas administrative concerns remained focused on the value of care despite resource limitations. From an industry perspective, attention was drawn to the investment required to implement AI tools and the willingness of administrators to support such adoption.

### Theme 3: Data Requirements

#### Patient-Centered Approach

All participants agreed that patient characteristics were the most critical factor in making a revascularization decision. The clinician participants indicated that making decisions around revascularization is particularly difficult for complex patients with multimorbidity.

Understanding and considering patient preference was another key factor in making revascularization decisions. The clinician participants indicated that giving patients a choice or following their preferences can be challenging, because the patient’s preference can sometimes be highly divergent from the evidence-based recommendation.

All participants agreed that respecting patient preference is important, and a conversation between the clinician and the patient, including hearing the patient’s perspective while educating patients about the risks and benefits of each intervention, should guide the decision-making process. It was noted that an AI-based CDSS could facilitate this discussion.

#### Evidence-Based AI

Participants stated that AI-based tools are expected to be based on evidence. The clinician participants emphasized that integrating existing evidence is important. They were intrigued by data standardization that could help AI to address real and predictable risks efficiently and consistently.

In addition, the clinician participants wondered if AI-based recommendations would differ from the current evidence and how they would reconcile such discrepancies. Despite this concern, they also expressed their excitement toward practicing data-driven revascularization decision-making.

#### Data Integration

Participants voiced the importance of integrating patient data into AI-based CDSSs. Participants stated that AI tools integrated with the electronic medical record could facilitate the clinical use of large volumes of patient data more efficiently and precisely. All participant groups also stated that integrating all critical patient data, including a comprehensive list of risk factors (eg, patient history, comorbidities, anatomical presentation, and frailty), is essential for AI-based CDSSs. Given the size and comprehensiveness of the data involved, some participants raised concerns regarding ethics, privacy, and confidentiality.

#### Contrasting Perspectives

All participants emphasized both the importance and the challenges of incorporating patient perspectives into revascularization decision-making. Clinicians suggested that an integrated AI-based CDSS may facilitate clinician-patient discussions and enhance decision-making; however, they also raised concerns regarding patient confidentiality and the potential unintended consequences of risk profiling that may adversely affect patients.

### Theme 4: Tool Characteristics

#### End User Built

All participants emphasized the importance of meaningfully involving all key end users when developing the tool. All participant groups stressed that end users should be involved not only in tool development but also in training, implementation, and evaluation. Furthermore, the patient and clinician participants noted that a patient-centered approach that captures patient voices and their perspectives about clinicians’ use of AI-based tools would lead to effective communication between patients and care providers. Some participants identified family physicians as potential end users as well.

#### Generation and Presentation of Decision Support Information

Participants were interested in the possibility that AI-based CDSSs have the capacity to provide a comprehensive score or recommendation that takes into account patient characteristics to optimize treatment plans and have the ability to interact with patients and families. Summary risk scores were preferred for easy interpretability and communication. Conversely, participants expressed concerns about the validity of the CDSS and including a comprehensive list of potential risk factors. Comprehensive data that integrated patient medical history and comorbidities were perceived as core components for successful AI-based CDSSs.

#### User-Friendliness and Accessibility

A user-friendly interface and being accessible beyond a networked computer (eg, mobile access) would increase uptake. In addition, participants stated that the CDSS needs to be intuitive and integrated seamlessly within the existing clinical workflow. Technical support would improve the usability and implementation of a CDSS, which would also address the concern about resources and workload associated with the tool (discussed in theme 2). The clinician participants also stressed that revascularization decisions require a high-level summary of accurate information, which must be easy to navigate and access.

#### System Logic, Reasoning, and Data Privacy

Most of the clinician and patient participants focused on the importance of the transparency of the CDSS and knowing how the CDSS works and generates treatment recommendations, including its logic and limitations.

Some participants also expressed their concern about regulatory compliance, liability, and accountability of the CDSS before implementation can be considered. This concern was based on medical ethics and legal perspectives. However, some participants also felt that if data privacy standards could be met, there was excitement about leveraging the advantages of AI.

Similarly, another major concern expressed by many participants was data privacy, particularly whether the AI-based CDSS could maintain the required confidentiality and privacy of health information. They pointed out that CDSS developers should be aware of various regulatory requirements that protect health information privacy. The patient participants were worried that AI recommendations could have unintended consequences on other health-related issues.

#### Contrasting Perspectives

Although all stakeholder groups agreed that AI-based CDSSs should be user-friendly, accessible, and developed with meaningful end user involvement, clinicians emphasized medico-legal considerations and the desire for such tools to support real-time decision-making and seamlessly integrate into clinical workflow. In contrast, patients may be more likely to view such tools as a means to facilitate discussion with care providers rather than solely functioning as a real-time decision aid. Both clinicians and patients emphasized the importance of validity and transparency of an AI-based CDSS; however, similar to theme 3, concerns remain regarding patient data privacy.

### Theme 5: AI Incorporation Into Clinical Workflow

#### AI as an Opportunity to Improve Care

Most participants perceived AI as an opportunity in the clinical workflow. They were quite positive and supportive about the development of AI-based CDSSs for revascularization decision-making. The clinician participants felt that AI would add support to their decision-making process, provided that recent scientific evidence is incorporated into the CDSS. Many participants mentioned that integration between the electronic medical record and AI, and end user engagement with both clinicians and patients, would be crucial to integration into the clinical workflow.

#### Knowledge Translation

Participants underlined knowledge translation as one of the core components required before integrating AI into the clinical workflow. For instance, the clinician participants were intrigued by how AI algorithms work, how comprehensive AI-based risk scores can be, as well as the system logic, reasoning, benefits, and limitations of the technology. However, many participants expressed that they still lacked knowledge about how AI works and suggested that continuous knowledge translation would be helpful.

Participants also noted that identifying end users is critical before integrating AI into the clinical workflow. For instance, a patient participant questioned whether AI-based CDSSs could be integrated into not only cardiac care but also primary care. The participant further expressed that integrating AI into primary care would facilitate conversations between patients and their primary care providers.

#### Contrasting Perspectives

While some clinicians emphasized the potential role of AI-based CDSSs as real-time supports within existing clinical workflows, others highlighted their value in supporting clinician-patient conversations across care settings. Differences in perspectives were most apparent in relation to knowledge translation, with clinicians emphasizing their desire for a deeper understanding of AI system functionality and validity, while other participants highlighted the importance of making AI-derived information simple and accessible to patients and primary care providers.

## Discussion

### Principal Results

This study provides an exploratory examination of AHS stakeholder perspectives on the use of an AI-based CDSS for CAD treatment strategy. Across stakeholder groups, participants emphasized that delivering evidence-based care when making coronary revascularization decisions for patients with CAD is often challenging due to conflicting or inadequate clinical practice guidelines. Although AI has the potential to improve the current state of CAD treatment decision-making, the need for timely access to comprehensive patient data through integration with hospital information systems, clinicians’ heavy workloads, data privacy concerns, and end users’ desire for transparency regarding how the AI generated a particular recommendation may complicate the successful development, deployment, and adoption of AI-based CDSSs. The likelihood of successful adoption can be enhanced by incorporating scientific evidence and patient preference into decision support information, involving end users (both patients and clinicians) in the entirety of technology development, designing systems that are intuitive and easy to use, and presenting information in a succinct and easy-to-understand manner. In addition, this study’s participants also raised the importance of systemic issues, including regulatory requirements, the need for resource commitments from health systems, and health care cost considerations.

Despite these challenges, the participants expressed excitement about AI’s potential to improve CAD care. Many acknowledged that they had limited AI knowledge and wanted to be educated in an ongoing manner. They felt that although substantial investments may be required to develop, implement, and adopt AI-based CDSSs, the potential cost savings and improved patient outcomes will likely make it a worthwhile endeavor.

### Contrasting Perspectives

Across themes, consistent patterns emerged in how different stakeholder groups framed the challenges and opportunities associated with AI-supported revascularization decision-making. Clinicians primarily focused on the practical realities of care delivery, emphasizing limitations in guideline clarity, time pressure, workload, and difficulties with synthesizing large amounts of data. Many described how an AI-based CDSS could help synthesize complex data, enhance clinical efficiency, and support real-time decision-making. Clinician concerns centered on liability and accountability of implementing AI-based tools, how to reconcile AI recommendations with clinical judgment, and emphasized the importance of patient data security and privacy. While clinicians often focus on mitigating long-term mortality and major adverse event risk, patients emphasized the importance of considering both short and long-term risks and benefits, valuing AI-based CDSS as a tool to help facilitate communication between clinicians and care providers. Industry perspectives highlighted the potential challenges surrounding an AI-based CDSS implementation, emphasizing the need for early alignment with administrative priorities, investment considerations, and demonstrable value to support adoption. Health system administrative perspectives, in contrast, tended to frame decision-making through a system-level lens, prioritizing standardization and value of care, viewing an AI-based CDSS as a short-term cost with potential long-term benefits and cost savings. Finally, differences were evident in expectations around knowledge translation, with clinicians seeking a deeper understanding of transparency, validation, and functionality of AI-based systems, while other participants emphasized the importance of making AI-derived information accessible to patients and primary care providers. Together, these contrasting perspectives highlight that the successful implementation of AI-based CDSSs requires not only technical accuracy but also careful alignment with the distinct priorities, responsibilities, and expectations of diverse stakeholder groups.

### Comparison With Prior Work

The potential of AI in improving CAD or cardiovascular care at large has previously been discussed [11,12], and a number of machine learning models have been developed in this space for a variety of clinical use cases (eg, in other studies [13-17]). Although much of this research has focused on the technical aspects of development, rather than implementation and adoption [18], many of the themes that emerged from the World Café are echoed in the existing literature. Challenges introduced by the availability, quality, and standardization of data are consistently raised in discussions surrounding the use of AI in health care [19-21], and concerns about data privacy and security are also common [22,23]. Many studies investigating clinician uptake of AI-based CDSSs emphasize the importance of providing evidence-based decision support information [21,24-27], using development data that are reflective of the patient populations the AI is intended to support [28-30], and increasing the transparency of model reasoning as important facilitators to adoption [22-24,31]. These studies also underline the issues resulting from an absence of AI education in the current medical curricula and call for increased knowledge translation efforts to build trust and credibility with clinicians [24,32]. It is worth noting that several issues discussed by the participants, including time constraints and the need for intuitive and user-friendly applications, are pervasive issues in health care that are not necessarily unique to the adoption of AI-based tools [33,34]. Indeed, with a greater than 50% failure rate among many types of CDSS [35], the need for end user involvement in development has been highlighted as crucial to successful implementation [35,36].

While many of the issues identified in this study appear to be universal across health care, contextual idiosyncrasies remain. For example, the WCPs often stressed the importance of patient preference in CAD treatment decision-making and suggested that an AI-based CDSS may help facilitate patient discussions. In contrast, some prior studies have reported concerns about AI-based CDSSs negatively impacting the patient-clinician relationship, suggesting that using such systems could reduce the amount of time available for patient interaction or, in extreme cases, cut out contact altogether should clinical interactions be shifted to a digital format [22,25]. However, one of these studies interviewed only general practitioners [25], whose responsibilities are arguably more conducive to digitization than coronary revascularization decision-making. Similarly, other studies have discussed technical barriers beyond data requirements, including insufficient computing resources and inconsistent access to Wi-Fi, which are more pervasive in low-to-middle-income countries [28,37]. Thus, the importance of this work also lies in understanding the context within which the AI-based CDSS will be implemented, which is essential for successful adoption [38-41].

Finally, although AI-based clinical tools are increasingly being evaluated for their potential to enhance clinical decision-making and efficiency [42], their utility should be met with both optimism and caution. Recent evidence suggests that while AI-based systems can accurately perform specific clinical tasks and support information delivery, these capabilities may not necessarily translate into improved patient or system-level outcomes [43,44]. Indeed, despite substantial investment and academic efforts, there remains limited prospective evidence demonstrating that AI-based tools have improved patient outcomes at scale [45]. Concerningly, among approved medical devices that use AI, clinical validation studies are inconsistently reported, and when reported, rarely include prospective and randomized evaluations [46,47]. Accordingly, while the technical performance of AI-based tools remains important, prospective randomized controlled trials are needed to demonstrate true clinical benefit. In parallel, future investigations should explicitly evaluate the fairness and equity of these tools and aim to determine whether such tools can alleviate clinician burden rather than add to cognitive or administrative loads [48].

We hope the diverse contextual factors described here can serve as a helpful foundation for the future development and implementation of CDSSs for CAD treatment decision-making.

### Limitations

This study has several limitations. First, 3 clinician participants were unable to attend the World Café, and they were engaged in one-on-one interviews instead. Although efforts were made to engage these participants using comparable prompts and facilitation techniques, inherent differences between one-on-one and group-based World Café discussions may have influenced the data generated. As a result, some themes may reflect methodological differences despite efforts to reconcile findings across formats. Second, within our relatively small sample size, clinicians outnumbered the other stakeholder groups (8 vs 4), and their opinions may have dominated the discussions and results. For instance, some patient perspectives were articulated by clinicians rather than patients directly and may not necessarily represent the views or priorities of the patients themselves. Third, all participants were recruited in Alberta, limiting the generalizability of our findings to other jurisdictions. As the overall structure of AHS, including the clinical flow of patients and delivery of care, may not mirror that of other health care systems, some of the themes identified in the current investigation may be unique to this publicly funded and integrated health care system. Accordingly, while the themes and perspectives derived from this investigation provide valuable insight into the intricacies of implementing AI-based CDSS into practice, future work incorporating larger and more heterogeneous participant populations, balanced stakeholder representation, consistent data collection formats, and diverse health care system contexts may provide additional insights.

### Conclusions

Various stakeholders, including patients and clinicians, believe that current coronary revascularization decision-making for patients with CAD is only partially evidence-based. AI-based CDSSs have the potential to improve this, leading to improved patient outcomes and health care cost savings. The successful development and implementation of such AI-based CDSSs hinges upon extensive end user involvement, data integration, data privacy protection, incorporation of patient preference, alignment with scientific evidence, and great usability. Integrating end user co-design and iterative usability testing may help support these priorities in future work.

## Supplementary material

10.2196/81303 Checklist 1 COREQ checklist.

## Abbreviations

AHS: Alberta Health Services
AI: artificial intelligence
CABG: coronary artery bypass grafting
CAD: coronary artery disease
CDSS: clinical decision support system
COREQ: Consolidated Criteria for Reporting Qualitative Research
OIP: one-on-one interview participant
WCP: World Café participant
